# Owner personality and the wellbeing of their cats share parallels with the parent-child relationship

**DOI:** 10.1371/journal.pone.0211862

**Published:** 2019-02-05

**Authors:** Lauren R. Finka, Joanna Ward, Mark J. Farnworth, Daniel S. Mills

**Affiliations:** 1 College of Life Sciences, Joseph Bank Laboratories, University of Lincoln, Lincoln, United Kingdom; 2 Animal, Rural and Environmental Sciences, Nottingham Trent University, Brackenhurst Campus, Southwell, United Kingdom; Universidade do Porto Instituto de Biologia Molecular e Celular, PORTUGAL

## Abstract

Human personality may substantially affect the nature of care provided to dependants. This link has been well researched in parents and children, however, relatively little is known about this dynamic with regards to humans’ relationships with non-human animals. Owner interactions with companion animals may provide valuable insight into the wider phenomenon of familial interactions, as owners usually adopt the role of primary caregiver and potentially surrogate parent. This study, using cats as an exemplar, explored the relationship between owner personality and the lifestyles to which cats are exposed. In addition, it explored owner personality as it related to reported cat behaviour and wellbeing. Cat owners (n = 3331) responded to an online survey examining their personality and the health, behaviour and management of their cats. Owner personality was measured using the Big Five Inventory (BFI) to assess: Agreeableness, Conscientiousness, Extroversion, Neuroticism and Openness. Owners also provided information concerning the physical health, breed type, management and behavioural styles of their cats. Generalised linear mixed models were used to identify relationships between owner personality and a range of factors that may have welfare implications for the wider companion animal population, and specifically, cats. Higher owner Neuroticism was associated with an increased likelihood of non-pedigree rather than pedigree cat ownership, a decreased likelihood of *ad libitum* access to the outdoors, cats being reported as having a ‘behavioural problem’, displaying more aggressive and anxious/fearful behavioural styles and more stress-related sickness behaviours, as well as having an ongoing medical condition and being overweight. Other owner personality traits were generally found to correlate more positively with various lifestyle, behaviour and welfare parameters. For example, higher owner Extroversion was associated with an increased likelihood that the cat would be provided *ad libitum* access to the outdoors; higher owner Agreeableness was associated with a higher level of owner reported satisfaction with their cat, and with a greater likelihood of owners reporting their cats as being of a normal weight. Finally higher owner Conscientiousness was associated with the cat displaying less anxious/fearful, aggressive, aloof/avoidant, but more gregarious behavioural styles. These findings demonstrate that the relationship between carer personality and the care received by a dependent, may extend beyond the human family to animal-owner relationships, with significant implications for the choice of management, behaviour and potentially the broader wellbeing of companion animals.

## Introduction

Familial relationships are key to the behavioural development and well-being of individuals. Exploration of other social roles, including that between humans and non-human animals may be able to cast light on animal experiences and welfare, but also act as a conduit for wider familial and relationship-based research. As a model, the associations between humans and non-human animals are of interest because the management of animals in captivity, irrespective of the purpose, relies upon the provision of care by humans, and generally includes a degree of social interaction. The nature of such care provision may have beneficial or detrimental impacts on animals’ physical and emotional wellbeing. However, the majority of this research has focused on the type of environment or management systems humans expose animals to, and the nature of the resources provided within them [1–7]. Much less consideration has been given to the impact of the human-animal relationship upon the animal. Handling and interaction styles have been shown to impact upon the behaviour, health and welfare of animals [8–19], where handler variations are potentially mediated by caretaker attitudes and their personality [9,17,18,20–23]. However, this latter effect has predominantly been explored in the context of agricultural animals, with an emphasis more on animal productivity than individual wellbeing per se [17,20–22]. Several studies have also explored this phenomenon in dogs [9,18,19,23], although the relationship between owner/caretaker personality and care outcomes for dogs were not measured, and in only one study were factors relevant to animal wellbeing directly assessed [19].

With increasing focus on the importance of individual differences amongst animals and the impact of such differences on their welfare within a given environment, there is also a need to consider such variations as important mediators or mitigators of wellbeing. For example, demographic features such as an animal’s age [24–26], breed type [27–29] and personality [30–34] may influence the extent to which individuals are at risk. Individual differences in animals such as their aesthetics [35–37], and behaviour [38] may also influence human’s perception of them and potentially play a role in their selection, purchase or adoption [36,39–41]. These differences may also determine outcomes for the selected animal including care provision, relinquishment [42–46] or even the decision to euthanize [43,47,48]. Aesthetics, in particular also determine selection for breeding and may drive breed variations associated with inherent and long-term health compromises [49–54].

The factors affecting the welfare of captive animals even within a given context are therefore, multifactorial and include characteristics of both the individual and their human caretaker. As companion animals are often managed on an individual basis, their selection and subsequent lifestyles are likely to be influenced by more than economic considerations, and incorporate individual caregiver preferences and personality. However, there is a relative paucity of research concerning the impact of such individual variation in relation to the quality of care delivered to animals, especially in a context where the carer may often believe the relationship is akin to that of the parent-child [55–58]. Such impacts are particularly pertinent to the welfare of companion animals where there are often frequent, prolonged periods of close control exerted by their caretakers, and for whom the experiences within such contexts are intrinsically linked to their social role within the household [59–63]. In most cases, strong and mutually compatible owner-companion animal bonds develop [55,64–66], but these do not necessarily ensure good welfare. For example, whilst most owners might believe that they provide a good standard of care, epidemic problems, such as obesity in cats and dogs [67–69] suggest this good intention does not always translate into effective practice.

The relationship between parent personality and emotionality and the development, behaviour and wellbeing of children is well established [70–74]. While 30–60% of personality traits are reportedly inherited [75], the remainder are considered to depend on psychosocial factors, including the nature of the child-parent relationship [76]. Human personality studies have shown that, in particular, the trait Neuroticism is strongly linked with negative outcomes for the individual. These include poorer physical and mental health, as well as generally lower quality of life [77], which may negatively impact on the well-being of those for whom these individuals are responsible. People scoring higher in Neuroticism may be more likely to show hostility and suffer from anxiety, anger, depressions, self-consciousness, impulsiveness and vulnerability [77]. The emotional instability of highly neurotic individuals may make them vulnerable to experiencing wide mood swings [78], stress and mental illness [79], as well as display maladaptive coping responses [80].

This tendency towards negative emotionality can also result in individuals applying negative intentions to the behaviour of others [81], and experiencing difficulty in maintaining positive emotional interactions [82]. Such characteristics may therefore create chaotic and unstable home environments, which may impact on the individuals around them, especially those with whom they have social relationships. Higher scores for parental neuroticism have been associated with less warm but more authoritarian and over protective parenting styles, which may result in the provision of more negative and intrusive parenting [83,84], a harshly controlled but poorly structured environment [85], or overly anxious concern for the child’s wellbeing [74]. Such types of parenting practices have been associated with behaviour problems in children including increased fighting, crying and whining [86], higher levels of shyness and emotional dysregulation [74], antisocial and aggressive behaviours [71], decreased empathy [81] and depression and anxiety [87]. In addition, evidence also suggests links between greater neurotic parental tendencies, childhood obesity [88] and lower general wellbeing in dependants [89].

Conversely, low parental Neuroticism but high Agreeableness, Conscientiousness, Openness and Extraversion have been associated with warmer parenting styles which provide more structured care and gentle control, less harsh discipline, less over protectiveness and more respect for the child’s autonomy [82]. Higher levels of parental Extraversion and Conscientiousness have also been associated with lower levels of behaviour problems in children [71,72] and higher levels of Agreeableness with less emotional dysregulation [74].

Research investigating pet owners’ personalities has tended to focus on the relationship between personality and pet preference or attachment style, or the complementarity of owner and pet personality and associated owner satisfaction [90–94]. The relationship between owner personality or emotionality and handling styles has also received some attention [9,18,19,95], although this has predominantly focused on dogs. Additionally, whilst inferences about the subsequent impact of owner personality on the wellbeing of their pets are made [94,96,97], parameters relating to the actual welfare of the animals are rarely applied or are less than conclusive. For example, equivocal evidence suggests that tense, shy, undisciplined and less emotionally stable owners are more likely to have dogs which behave aggressively [98], as well as display various other ‘problem behaviours’ [19], but also that dogs owned by people scoring higher in neuroticism (i.e. emotional instability [99]) have comparatively lower cortisol levels, potentially suggesting less stress [100,101].

Caretaker/owner personality traits that are beneficial in one species may not be so in others. For example, whilst in 2010 Wedl et al found that the dogs of more neurotic owners spent greater time in their proximity [102], similar observations in cats found that those with more neurotic owners chose to interact with them less [95]. The domestic cat is now one of the most common companion animals globally. In the United Kingdom alone, recent estimates suggest there are between 8 and 11 million pet cats [103–105]. Cats are subjected to a range of different types of management by humans (e.g. housed strictly indoors, given outdoor access, housed with other animals or individually, exposed to quiet or busy human households). They may also have very different types of relationships with humans which vary in the degree of autonomy given to the cat (e.g. treated as a social companion or simply kept for external pest control, treated as a ‘fur baby’ [56] or expected to be relatively independent. Such relationships may result in the cat partaking in mutually beneficial, affiliative interactions, or merely tolerating social proximity due to the associated physical resources provided [106]. These factors may differentially impact on the wellbeing of the cat, depending on the individual in question [107–112]. The domestic cat maintains the potential for variable and flexible social organisation, potentially occupying a range of different lifestyles with varying degrees of human social contact [113,114], often existing upon a spectrum of sociability towards people [47,106,115,116]. Some environments and caretaker profiles may be particularly challenging for a species which has become domesticated only relatively recently and may still be transitioning to a close domestic cohabitation with humans [117–121].

**Fig 1:**
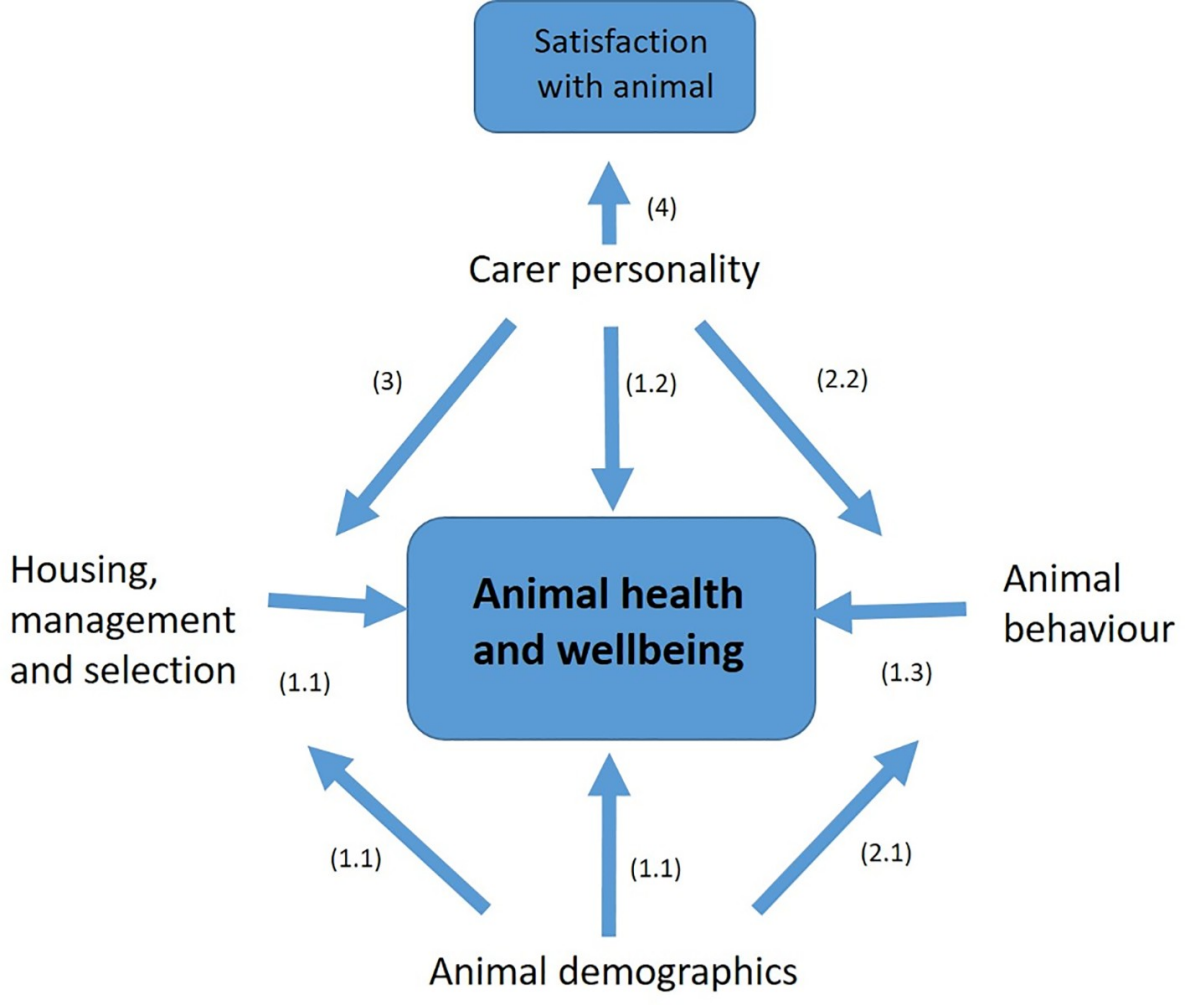
Animal wellbeing and human management. Illustration of the potential interactions between factors anticipated to be relevant to the wellbeing of animals managed or cared for by humans. Each of the main hypotheses are mapped numerically according to the text below, against the relevant arrow to indicate the anticipated relationship between factors, and their directionality.

The aim of this study was therefore to elucidate potential relationships between the personality of the caretaker, and the lifestyles, behaviour and wellbeing of their cats. The complexity of this relationship is illustrated in Fig 1 and forms the basis of the main hypotheses of our research, which were split into four main areas.

The primary hypotheses were that:

1. Proxy measures of cat welfare (e.g. cat weight, composite scores for stress-linked sickness behaviours and house soiling) are affected by:
  1.1 The lifestyle and demographic features of the cat (e.g. their age, breed, presence of an existing medical condition and type of outdoor access they are provided with)
  1.2 The personality traits of their owner
  1.3 The general behavioural style of the cat. We define ‘behavioural style’ as the tendency of the cat to behave in a certain way within given situations; in this case in the context of human interactions.
2. The general behavioural style of the cats is affected by:
  2.1 The demographics of the cat
  2.2 The personality traits of their owner
3. The selection of cats and their management is affected by the personality traits of the owner
4. The satisfaction of owners with their cats and relinquishment consideration is affected by the personality traits of the owner

## General methods

### Questionnaire development

The questionnaire comprised of six sections (see S1 Appendix for the full questionnaire and options provided). Where individuals owned more than one cat, they were asked to select a focal individual.

- Household demographics: These included items about the person’s gender, number of cats owned, method of focal cat acquisition and type of outdoor access provided to the focal cat.
- Owner satisfaction and thoughts on relinquishment, including questions rated on a 5-point Likert scale concerning personal satisfaction with the focal cat, and the frequency with which they had considered its relinquishment.
- Owner personality: This used the 44 item Big Five Inventory [77] which is one of the most well validated survey-based measures of human personality [77,99,122–124]. It also provides a practical means by which to assess key trait dimensions via self-reporting. Each trait was assessed via a composite score based on participant agreement with various statements about themselves, rated on a 5-point Likert scale.
- Focal cat demographics including age, breed (whether pedigree or non-pedigree), and neuter status.
- Focal cat health and wellbeing questions including:
  - Whether the cat had an existing medical condition (yes/no with an open response option available).
  - A series of questions rated on a 5-point Likert scale explored the cat’s coat condition and the frequency of stress-related sickness behaviours, including those related to cystitis, vomiting, diarrhoea and constipation [108,125,126].
  - A series of questions rated on a 5-point Likert scale concerning the frequency of behaviours relating to house soiling including defecation and urination outside of the litter tray and spraying on vertical surfaces indoors [111].
  - A rating of the cat’s weight category (scored as normal, overweight, very overweight, underweight or very underweight).
- Focal cat problem behaviour and behavioural styles including:
  - The presence of a ‘behavioural problem’ in the cat (recorded as yes/no with a response box provided to allow indication of the behaviour).
  - A series of 28 questions regarding the cat’s behaviour, primarily towards the owner, rated on a 5-point Likert scale representing agreement with statements about the cat. Items were derived from a survey developed by Finka in 2015 [127] based on their hypothesised link to friendliness, fearfulness and frustration, and associated affective systems (sensu Panksepp [128]). All items had previously demonstrated longitudinal intra-rater repeatability when tested within a large population of cat-owners over a 6-month period.

Ethical approval:

Ethical approval for this project was granted by the University of Lincoln and Nottingham Trent University (ref: ARE820).

### Data collection

The survey was available through Survey Monkey from June-July of 2016. A convenience self-selecting sample of cat owners was recruited via social media sharing and posting to cat-based pages, sharing on International Cat Care’s website, and poster advertisements in veterinary surgeries in South West London. Participants were informed that the survey aimed to investigate owner personality and the cat-human relationship. Participants were required to be 18 years or older and have lived with their cat for at least six months. Individuals owning more than one cat were instructed to select the cat they felt they knew the best. Participants were advised that by completing the questionnaire, they were giving their permission to participate in the study. They were informed that any information provided would be held in accordance with the University of Lincoln’s data protection regulations and only used for the purposes of the study. Participants were given the option to provide their email address if they wanted to receive a summary of the results from the questionnaire, however all data were stored and analysed anonymously.

### Data analysis and statistical methods

Composite scores were generated for the cat’s frequency of sickness and house soiling behaviours, cat’s behavioural style (following principal component analysis), owner satisfaction and owner personality traits. All other variables (see S1–S4 Appendices for composite structure) were either categorical (i.e. cat outdoor access type, breed type, weight category, presence of a behavioural problem or existing medical condition, neuter status, owner’s age, cat and owner gender) or were counts based on single measures (i.e. frequency of relinquishment consideration, cat’s age).

The analyses addressed the primary hypotheses of the research (see Fig 1). Firstly, the underlying relationship between the various health and welfare parameters and cat lifestyle and demographics (1.1) was assessed. Results of this initial stage then informed which factors to include as covariates in further analyses of cat health and welfare parameters relative to owner personality (1.2) and the behavioural styles of the cat (1.3). The next phases involved assessing the relationship between the behavioural style of the cat and both cat demographics (2.1) and personality of the owner (2.2). Finally, the relationships between owner personality and owner selection and management of cats (3) and owner satisfaction (4) were explored.

#### Cat welfare, lifestyle and demographics

The relationship between proxy measures of cat welfare and demographic features of the cat were assessed via Generalised Linear Models (GLMs). In separate analyses, the cat’s sickness behaviour and house soiling score were included as the response variable, and the cat’s age, presence of a medical condition (yes/no), breed (pedigree or non-pedigree), and their interaction included as explanatories in both cases. In an additional analysis, whether the cat had an existing medical condition was included as the response variable, and the cat’s age and breed and their interaction were included as the explanatories.

The relationship between cat’s sickness behaviour scores (included as the response variable) and the type of outdoor access they were provided with (either kept strictly indoors, given restricted or unrestricted outdoor access, included as the explanatories), was then assessed via Generalised Linear Mixed Models (GLMMs), with both the cat’s age and the presence of a medical condition included as random effects (due to their significant relationship with the response variable, as identified in the preceding analysis).

The following relationships between demographic features of the cat and their lifestyles were then assessed via a series of GLMs to determine:

- The relationship between the type of outdoor access provided (included as the response variable) and the cat’s breed type (included as the explanatory)
- The relationship between the cat’s reported weight status (included as the response variable) and the cat’s breed and outdoor access type, and their interaction (included as the explanatories).

#### Cat welfare and owner personality

The relationship between owner-reported cat health parameters and owner personality were assessed via a series of separate GLMs and GLMMs which included the following:

- The presence or absence of a pre-existing medical condition in the cat (included as the response variable), and the five owner personality trait scores (included as the explanatories), with the cat’s age and breed included as random effects (due to their significant relationship with the response variable, as identified in the previous analyses)
- The cat’s sickness behaviour score (included as the response variable), and the five owner personality trait scores (included as the explanatories), with both the presence of a medical condition and type of outdoor access provided included as random effects (due to their significant relationships with the response variable, as identified in the previous analyses)
- The cat’s house soiling score (included as the response variable), and the five owner personality trait scores included as the explanatories, with the cat’s age included as a random effect (due to its significant relationship with the response variable, as identified in the previous analyses)
- The cat’s weight category, (included as the response variable), and the five owner personality trait scores included as the explanatories, with the cat’s breed type included as a random effect (due to its significant relationship with the response variable, as identified in the previous analyses)

#### Cat welfare and behavioural style

In order to identify key behavioural styles based on the similarity and dissimilarity of individual items, the 28 items relating to the cat’s behaviour towards the owner were subjected to a Principal Component Analysis (PCA). Retained principal components were then interpreted and used to generate scores for each cat on each composite behavioural component by adding together the scores of retained items (see statistical methodology for details of criteria used).

The relationship between proxy measures of cat welfare and the cat’s behavioural styles were assessed using GLMMs. In separate analyses, the cat’s sickness behaviour score and house soiling score were included as the response variable, and the cat’s Principal Component (PC) scores and their interaction included as the explanatories. Age of the cat was included as a random effect in both analyses (due to its significant relationship with the response variable, as identified in the previous analyses), with owner’s Neuroticism scores included as an additional random effect in the analyses focusing on sickness behaviour scores (due to its significant relationship with the response variable, as identified in the previous analyses).

#### Cat behavioural style and breed

The relationship between the cat’s behavioural style and their breed type was assessed using a series of GLMs. In separate models, cat’s PC scores for each of the main behavioural PC components (based on the preceeding analysis) were included as the response variables with cat’s breed type included as the explanatory.

#### Cat behaviour and owner personality

The relationship between the cat’s behavioural style and the personality of the owner was then assessed via a series of GLMMs. In separate models, cat’s PC scores for each of the extracted PC components were included as the response variables, with the five owner personality trait scores included as explanatories, and the cat’s breed included as a random effect (due to its significant relationship with the response variable, as identified in the previous analyses).

The relationship between the cat being reported as having a ‘behaviour problem’ and the personality of the owner was assessed via a GLM with the presence of a ‘behaviour problem’ included as the response and the five owner personality trait scores included as explanatories.

#### Cat selection, management and owner personality

Owner selection methods and preferences and their relationship with owner personality were assessed via a series of separate GLMs. In each respective model, source of cat acquisition, number of cats owned, and breed type were included as the response variable with the five owner personality trait scores included as the explanatories.

The relationship between the type of outdoor access provided to the cat (included as the response variable) and the five owner personality trait scores (included as the explanatories) was assessed using a GLMM, with breed included as a random effect (due to its significant relationship with the response variable, as identified in the previous analyses).

#### Owner satisfaction, relinquishment and owner personality

Owner satisfaction and frequency of the owner considering relinquishing their cat were assessed for their relationship with owner personality scores. In separate GLMs, satisfaction scores and relinquishment ratings were included as the response variable and the five owner personality trait scores as the explanatories.

### Statistical methodology

All statistical analyses were carried out in R version 3.4.2 [129].

For both GLMs and GLMMs, the relationship between the explanatory and response variables were analysed via backwards deletion of fixed effects to reach a minimum adequate model [130] using package lme4 with maximum likelihood fits for the GLMMs [131]). Depending on the nature of the response variable (i.e. continuous or categorical), either Poisson or Binomial family structures were included. Where response variables were categorical and included more than two levels, a series of separate post hoc models including only 2 levels each time were run in order to target the direction of effects. Where analyses included multiple comparisons of fixed effects on the same response variable, Bonferroni corrections were applied [132]. Model diagnostics were performed to assess normality, heteroscedasticity and check for overdispersion.

For the Principal Component Analysis, PC’s were extracted from the correlation matrix and subject to a varimax rotation using the *principal* function within the *psych* package [133]. All variables were based on the same scale and were thus not subjected to rescaling prior to analyses. The scree plot, Eigen values and percentage of variation explained for each component were examined and used to determine the number of components to retain. Item loadings of ≥ |0.4| on each of the retained components were used for the purposes of component interpretation, based on the nature of the items and their direction of loading (either positive or negative). On this basis, four items were used to generate component scores for each cat for each of the four principal components identified. See S2 Appendix for a list of the retained items in each component.

## Results

### Cat owner demographics (for full details see S3 Appendix)

A total of 3331 individuals responded to the questionnaire with 3165 (95%) completing the survey in its entirety and providing viable responses (i.e. not obviously duplicates or answering questions for more than one cat at once; identified based on responses to open ended questions). The vast majority of respondents were female (2923, 92%). Most of the population fell into one of three age categories; 25–35 and 35–44 (both 25.9%), and 45–54 (23.3%).

#### Cat ownership, acquisition, satisfaction and relinquishment

The number of additional cats, excepting the one the owner was completing the survey for, ranged from 0 to 32, with a median of 1 and average of 1.4 (± 2.2sd). More people acquired their cat from a rescue/rehoming/foster network than any other source (1098, 34.7%), with the next most popular source being a friend/family member/ acquaintance (825, 26.1%).

Average satisfaction scores were very high at 14.12 (± sd 1.5, scale range: 5–15, min: 3 max: 5). The median frequency score for considering relinquishment was “never” (score 1 on the scale range 1–5, min: 1 max: 5).

#### Owner personality

Average scale scores for the 5 personality traits were: Agreeableness 3.85 (± sd 0.61), Conscientiousness 3.631 (± sd 0.43), Extroversion 3.22 (± sd 0.61), Openness 3.75 (± sd 5.8), and Neuroticism 3.01 (±sd 0.84).

### Cat demographics (for full details see S4 Appendix)

#### Breed, age and neuter status

The majority of cats (2612, 82.5%) were non-pedigrees such as domestic long, medium or short hair, with the remainder comprising different pedigrees and their crosses. The vast majority of cats were neutered (3077, 92.7%) and the average age was 7.194 years (± sd 4.9), ranging from ≤1 to 24.

#### Health and house soiling

The majority of owners (2423, 76.6%) reported that their cat didn’t have a medical condition, with the remainder reporting a range of predominantly chronic conditions including diseases associated with internal organs, tumours, joints and cognitive problems. The average sickness behaviour score (with 25 representing the best health) was 22.11 (± sd 2, scale range: 5–25, min: 14, max: 25). Average house soiling score (with 15 representing a total absence of house soiling) was 14 (± sd 1.6, scale range: 5–15, min: 3, max: 15). The majority of cats (2401, 76%) were rated as being of a normal weight, with 552 (17%) being rated as over overweight or very overweight, and 211 (7%) rated as underweight or very underweight.

#### Outdoor access provision

It was most common for cats to have access to the outdoors which was restricted in some way (i.e. the cat is kept in at night or is only let out when someone is home), (1423, 45%). Providing the cat with unrestricted outdoor access or keeping them strictly indoors was less common (916, 28.9%) and (826, 26.1%) respectively.

#### Cat problem behaviour and behavioural styles

Only 623 people (20%) indicated that their cat exhibited a behavioural problem. These included a diverse range of behaviours such as aggression, pica, house soiling, scratching on the furniture and attention seeking (see S4 Appendix for full list).

PC eigenvalues indicated four main components which explained between 23 and 5% of the variation, with eigenvalues between 6.5 and 1.9. The four extracted components contained between ten and five items, which loaded at or above the specified threshold. Ten items were retained within the first PC. These included a range of items relating to the friendliness and boldness of the cat, and this PC was labelled as ‘Gregariousness’. Seven items were retained within the second PC. These included items relating to agonistic styles of behaviour and a lack of handling tolerance, and this PC was labelled as ‘Aggressiveness’. Eight items were retained within the third PC. These included items relating to avoidant and unfriendly styles of behaviour, and this PC was labelled as ‘Aloofness/avoidance’. Five items were retained within the fourth PC. These included items relating to anxious and fearful styles of behaviour, and this PC was labelled ‘Anxiousness/fearfulness’.

Average scores for the ‘Gregariousness’ PC1 were 37.8 (± 6.4sd, scale range: 10–50, min: 12, max: 50), for the ‘Aggressiveness’ PC2 were 11.32 (± 4 sd, scale range: 7–35, min: 7, max: 33), for the ‘Aloofness/avoidance’ PC3 were 15.22 (± 4.6 sd, scale range: 8–40, min: 8, max: 39) and for the ‘Anxiousness/fearfulness’ PC4 were 15.98 (± 3.7 sd, scale range: 5–25, min: 5, max: 25).

### Cat welfare parameters and lifestyle (for full details of statistical outputs see S6 Appendix)

#### Cat breed, age, sickness behaviours and house soiling

Composite sickness behaviour scores were significantly associated with both age and the presence of an existing medical condition, but not their interaction. Older cats (z = -7.128, p<0.0001) and cats with a medical condition (z = -5.192, p<0.0001) had significantly lower sickness behaviour scores (indicating a greater frequency of sickness behaviours). There was no significant relationship between sickness scores and breed p>0.05).

The presence of an existing medical condition was significantly associated with both age and breed, but not their interaction. Pedigree cats (z = 2.14, p<0.05) and older cats (z = 16.91, p<0.0001) were significantly more likely to have an existing medical condition.

House soiling scores were significantly associated with age, with older cats having lower house soiling scores (z = -3.93, p<0.0001), therefore a higher reported frequency of house soiling. There was no significant relationship between house soiling and the presence of an existing medical condition or breed (p>0.05).

#### Outdoor access type, breed, weight and sickness behaviours

With both age and the presence of a medical condition included as random effects, sickness behaviour scores were significantly associated with outdoor access type. Sickness behaviour scores were significantly lower (indicating a higher frequency of sickness behaviours) among cats that were kept strictly indoors (z = -1.981, p<0.005) or had restricted outdoor access (z = -2.213, p<0.05), compared with those given unrestricted access to the outdoors. There was no difference in sickness scores between cats that were kept strictly indoors and those that had restricted outdoor access p>0.05.

The type of outdoor access provided to the cat varied significantly with breed. Compared to non-pedigree type cats (i.e. domestic short/medium/long hair), pedigree cats (all other breeds and clearly defined crosses) were significantly less likely to be given unrestricted (z = -9.504, p<0.0001) or restricted (z = -6.442, p<0.0001) access to the outdoors than be kept strictly indoors. They were also significantly less likely to be given unrestricted access than restricted access (z = -4.633, p<0.0001).

The cat’s weight category was significantly associated with breed (either pedigree or non-pedigree) but not outdoor access type (unrestricted, restricted access or strictly indoors) (p>0.05). Compared to non-pedigree cats, pedigrees were significantly more likely to be reported as being overweight than underweight (z = 5.454 p<0.0001), although no other comparisons between weight categories were significant (p>0.05).

### Cat welfare and owner personality (for full details of statistical outputs see S6 Appendix)

#### Existing medical conditions, weight, sickness behaviour and house soiling scores

With age included as a random effect, cats that were reported as having an existing medical condition had owners with significantly higher scores for Neuroticism (z = 3.982, p<0.0001). None of the other owner personality traits demonstrated a significant relationship with the cat having a medical condition (all p>0.05).

No significant relationship was detected between the cat’s house soiling score and any of the owner personality traits (all p>0.05).

With age, the presence of an existing medical condition, as well as the type of outdoor access the cat had (i.e. strictly indoors, restricted or unrestricted outdoors) included as random effects, higher cat sickness behaviour scores (indicating a lower frequency of sickness behaviours) were significantly associated with lower scores for owner Neuroticism (z = -2.692, p<0.01). None of the other personality traits demonstrated a significant relationship with sickness scores (all p>0.05).

With the cat’s breed included a random effect, reported weight category was significantly associated with the owner’s personality. Differences between weight categories indicated that owners who reported that their cats were either overweight or very overweight rather than a normal weight, had significantly lower scores for Agreeableness (z = -3.293, p<0.001), but higher scores for Neuroticism (z = 2.829, p<0.01). In addition, owners who reported their cats as being underweight or very underweight rather than of a normal weight had significantly lower scores for Agreeableness (z = -3.316, p<0.001), but higher scores for Extroversion (z = 2.112, p<0.05).

### Cat behavioural styles and welfare

With both the cat’s age, the presence of a medical condition, outdoor access type and owner’s Neuroticism scores included as random effects, none of the cat’s PC behaviour scores (i.e. ‘Gregariousness’–PC1, ‘Aggressiveness’–PC2, ‘Aloofness/avoidance’–PC3,’ Anxiousness/Fearfulness’–PC4) were significantly associated with cat’s sickness behaviour scores, either as individual factors or as interactions (p>0.05). No significant relationship was found between any of the cat’s PC and house soiling scores (p>0.05).

### Cat behavioural style and breed (for full details of statistical outputs see S6 Appendix)

The cat’s PC scores varied significantly based on their breed type (either pedigree or non-pedigree). Pedigree cats scored significantly higher on the ‘Gregariousness’ PC1 component (z = 11.27, p<0.0001), and significantly lower on the ‘Aggressiveness’ PC2 (z = -8.067, p<0.0001), ‘Aloofness/Avoidant’ PC3 (z = -7.645 p<0.0001) and ‘Anxiousness/Fearfulness’ PC4 components (z = -7.558, p<0.0001).

### Cat behaviour and owner personality (for full details of statistical outputs see S6 Appendix)

#### Cat behavioural styles and owner personality

Cat breed was included as a random effect within the analyses in this section.

PC1: Cat ‘Gregariousness’: Owner personality scores were significantly associated with cat’s scores on the component labelled ‘Gregariousness’. Higher cat ‘Gregariousness’ scores were associated with significantly higher owner Extroversion (z = 2.283, p<0.00001), Openness (z = 5.095, p<0.00001), and Conscientiousness (z = 5.154, p<0.00001) scores.

PC2: Cat ‘Aggressiveness’: Owner personality scores were significantly associated with cat’s scores on the component labelled ‘Aggressiveness’. Higher cat ‘Aggressiveness’ scores were associated with significantly higher owner Neuroticism scores (z = 2.277, p<0.05) but lower owner Agreeableness (z = -3.506, p<0.0001), Openness (z = -2.1, p<0.05) and Conscientiousness (z = -2.693, p<0.001) scores.

PC3: Cat ‘Aloofness/avoidance’: Owner personality scores were significantly associated with cat’s scores on the component labelled ‘Aloofness/avoidance’. Higher cat ‘Aloofness/avoidance’ scores were associated with significantly lower owner Agreeableness (z = -4.561, p<0.0001), Openness (z = -4.199, p<0.0001) and Conscientiousness (z = -5.062, p<0.0001) scores.

PC4: Cat ‘Anxiousness/fearfulness’: Owner personality scores were significantly associated with cat’s scores on the component labelled ‘Anxiousness/fearfulness’. Higher cat ‘Anxiousness/fearfulness’ were associated with significantly higher owner Neuroticism (z = 3.256, p<0.01) but lower owner Conscientiousness (z = -2.951, p<0.01) scores.

#### Cat behavioural problems and owner personality

Whether or not owners reported their cat as having a behaviour problem was significantly associated with the owner’s personality. Owners of cats with a reported behaviour problem had significantly higher scores for the trait Neuroticism (z = 2.383, p<0.05). No significant relationship between the other owner personality traits and the cat having a reported behaviour problem were apparent (all p>0.05).

### Cat selection, management and owner personality (for full details of statistical outputs see S6 Appendix)

#### Cat acquisition

None of the owner personality scores were significantly associated with the source of cat acquisition (all p>0.05).

#### Number of cats owned

Owner personality traits were significantly associated with the number of additional cats owned. Higher numbers of additional cats owned were associated with significantly lower scores for owner Extroversion (z = -6.817, p<0.0001), Agreeableness (z = -2.454, p<0.05) and Neuroticism (z = -2.780, p<0.01) but higher scores for Conscientiousness (z = 3.741, p<0.001) and Openness (z = 2.67, p<0.01).

#### Cat breed

Cat breed type was significantly associated with owner personality. Owner Neuroticism scores were significantly lower for people that reported that their cat was a pedigree rather than a non-pedigree (see S4 Appendix for the full list of breed types reported), (z = -4.764, p<0.0001). No significant relationships between the other owner personality traits and cat breed type were apparent (all p>0.05).

#### Provision of outdoor access

With breed included as a random effect, owner personality scores varied significantly based on the type of outdoor access the cat had. Comparisons indicated that:

- Owners that allowed their cat unrestricted access to the outdoors had significantly higher Extroversion scores (z = 4.441, p<0.001), and significantly lower Neuroticism (z = -2.871, p<0.01) and Openness scores (z = -2.783, p<0.01), compared to owners who kept their cats strictly indoors.
- Owners that allowed their cat unrestricted access to the outdoors had significantly lower scores for Neuroticism (z = -3.202, p<0.05) and also Agreeableness (z = -2.424, p<0.05), compared to owners that allowed their cats restricted access to the outdoors.
- Owners that allowed their cat restricted access to the outdoors had significantly higher Extroversion (z = 5.321, p<0.001) scores compared with owners that kept their cats strictly indoors.

### Owner satisfaction, relinquishment and owner personality (for full details of statistical outputs see S6 Appendix)

Owner satisfaction scores were significantly associated with owner personality. Owners with higher Agreeableness scores had significantly higher composite satisfaction scores (z = 2.359, p<0.05). No significant relationships between owner satisfaction and the other owner personality traits were apparent (all p>0.05).

No significant relationships between the frequency with which the owner considered relinquishing their cat and owner personality were apparent (all p>0.05).

## Discussion

Our primary aims were to elucidate potential relationships between the personality of caretakers and various aspects of their cat’s lifestyles and wellbeing, and to assess the degree to which these relationships mirrored those of the parent-child. Human personality traits are considered relatively stable/enduring traits [134] and so should not be affected by relatively proximate circumstances such as the cat’s behaviour. Thus, although we cannot be sure of the causal nature of the statistical relationships identified, we suggest that owner personality is perhaps more likely to underpin the more fluid differences in management, behaviour and welfare of the cats reported here. Our results identified a substantial number of such relationships, which were generally consistent in their direction across each of the four main areas relevant to our hypotheses. Our key findings were that higher owner scores for Neuroticism were significantly related to a number of factors relevant to the management, behaviour and health of cats, potentially indicating a link between owner personality and poorer welfare outcomes. Higher owner scores for Neuroticism were associated with a greater likelihood that the cat was a non-pedigree and would have either restricted or no outdoor access. More neurotic owners also reported more incidences of on-going medical conditions in their cats, that these cats were overweight or very overweight, displayed more frequent stress-linked sickness behaviours, ‘behavioural problems’ and aggressive or anxious/fearful behavioural styles.

At the same time, higher owner scores for Agreeableness were generally associated with more positive wellbeing outcomes for their cats. More agreeable owners were more likely to indicate that their cats had a normal weight and displayed less aggressive and aloof/avoidant behaviours. In addition, they also reported being more satisfied with their cat. Higher owner Extroversion, Conscientiousness and Openness were also generally associated with more positive aspects of the cat’s behaviour and wellbeing. More Extroverted owners were more likely to provide their cats with unrestricted access to the outdoors than keep them strictly doors, although interestingly the reverse was true for owners higher in Openness, who were more likely to keep their cats strictly indoors. In addition owners higher in Agreeableness were more likely to give their cats restricted rather than unrestricted access to the outdoors. Owners higher in Conscientiousness and Openness were more likely to report their cats as being more gregarious, but less aggressive and less aloof/avoidant. Owners higher in Conscientious also reported less anxious/fearful behavioural styles in their cats.

Our findings mirror the findings of research on parental personality, parenting styles and child behaviour in various ways. The fact that owners with higher Neuroticism scores were more likely to keep their cats indoors or restrict their outdoor access may reflect a generally more over-protective, overly anxious caretaking style; a link previously identified within the parent-child literature [74,135], with similar inferences also being made in dogs [19]. More Neurotic cat owners may be more concerned with risks to their cats whilst wandering outdoors, and thus restrict access in some way, protecting them from perceived threats [135]. However, we must acknowledge that this survey predominantly sampled a UK cat-owning population, and cultural differences may exist. In the UK, providing outdoor access for cats is commonplace [136,137], whereas in other parts of the world such as the in the US, keeping cats strictly indoors is more prevalent, and in some cases recommended by veterinary professionals, animal welfare organisations and conservationists [107,136,138–141]. Cultural norms may mask caretaker personality effects on the decision to keep a cat indoors, where those cultural factors are pervasive. Exploration of the interaction between owner personality, country of residence and cat management practices would be worth further investigation.

Diagnosis of ill health can be challenging for the average owner due to a tendency for cats to suppress many behavioural signs of pain and discomfort [142–145]. The overly anxious and concerned caretaker-style typically exhibited by more neurotic owners [74,135] may explain the greater prevalence of existing medical conditions and sickness behaviours reported by more neurotic owners in our study. Such owners may be more worried about, and thus observant of, their cat’s physical condition and signs of ill health. In turn, intervention and diagnosis rates may be increased through more rapid assessment. Owner-reported differences in cat health may also emerge from more neurotic owners having more negative or pessimistic views of their cat’s health, leading to over reporting of sickness behaviours, medical conditions, and misdescribing of the cat’s weight. Such perceptions however, might still be detrimental to the cat’s wellbeing if they result in frequent but unnecessary and inherently stressful visits to the veterinary clinic [146,147], or over-estimation of the cat’s weight leads to unnecessary dietary changes or food restrictions imposed upon them. However, the parent-child literature indicates a real link between higher parental Neuroticism and greater child obesity [88]. This likely suggests that the cats of more neurotic owners may actually be overweight. Additionally, in the human literature, more authoritarian parenting styles were found to correlate with greater child distress and lower general wellbeing [87]. The greater prevalence of poorer health and stress-linked behaviours reported for cats owned by more neurotic owners could therefore, be a direct consequence of the caretaker styles (i.e. the way the cat is generally managed and interacted with) to which cats are exposed. More neurotic owners may provide a more authoritarian, unpredictable, intrusive and or harshly controlled caretaker style [74,83–85] which could lead to greater levels of anxiety, stress and compromised immune function in their cats. Indeed, evidence suggests that exposure of cats to such types of harsh and unpredictable caretaking leads to greater behavioural and physiological signs of stress [39,108,125,148].

In the current study, owner personality was only assessed relative to cat management concerning outdoor access, rather than resource provision more generally (e.g. overall quality and quantity of indoor and outdoor resources provided), or more holistic aspects of caretaker style (e.g. that encompass the owner’s general behavioural style towards the cat). Evidence does suggest that owner personality affects interpersonal behaviour [149] and that, in the short-term, cats’ interactions with more neurotic owners suggest less active participation and thus more avoidance from the cat [95]. However, in dogs, short-term assessments of owner’s behaviour towards their dogs found no relationship between owner-dog interaction styles and owner Neuroticism [18]. In another study, differences were found between the structures of owner’s ‘dog-directed’ parenting styles and those of ‘child directed’ parenting styles [150]. Therefore the relationship between parenting/caretaker styles and caretaker personality in the parent-child dynamic may not necessarily be replicated in the owner-pet dynamic. Further research is thus required to explore how owner personality directly relates to general pet resource provision, owner-cat interactions across various contexts (i.e. during both tactile and non-tactile interactions, when feeding the cat or providing access to rooms/the outdoors), as well as more general caretaker/parenting styles (i.e. authoritative, authoritarian, permissive), and how these in turn impact upon the behaviour and welfare of cats over longer and unstructured time periods.

As cats cannot self-report, the behavioural and physical indicators of stress surveyed in this study were used to provide practical, proxy measures of basic wellbeing. However, such measures are no substitute for direct behavioural observations and the collection of suitable biological data that may better quantify aspects of cat welfare.

Higher owner Neuroticism was also associated with greater reports of cat ‘behaviour problems’ and a greater expression of negative cat behavioural styles. Meanwhile, higher owner Agreeableness, Conscientiousness and Openness were generally associated with lower occurrences of these behaviours. With the exception of Extraversion, a similar trend was previously identified in dogs in relation to owner personality traits and prevalence of dog ‘problem behaviours’ such as owner-directed aggression, stranger-directed fear and/or house soiling when left alone [19].

Again, such relationships also parallel findings within the parent-child literature. In humans, higher parental Neuroticism and lower levels of Agreeableness and Conscientiousness have been associated with overt antisocial and aggressive behaviours [71] and more defiance, anger and behavioural problems [151]. Contrastingly, higher levels of Conscientiousness [72] and lower levels of Neuroticism correlate with fewer child externalising behaviours [72,152] and delinquency [153]. Similar to sickness behaviours, cats’ more negative behavioural responses when owned by more neurotic individuals could be in direct response to such owners displaying more hostile, less warm and unpredictable styles of caretaking. Such experiences are likely to increase the stress levels of cats and reduce their sense of social and/or environmental control. As a result, this may cause anxiety and/or frustration and lead to avoidant and aggressive styles of behaviour, depending upon the individual cat and the context [154].

The higher levels of anxious and aggressive behaviour reported for cats owned by those with higher Neuroticism scores might also be due to owner misattribution of negative behavioural intentions to their cats [81]. If this is the case however, our results suggest that this does not significantly negatively impact on their level of satisfaction with the cat or the frequency with which they consider relinquishment. Indeed, other research suggests neuroticism to be positively correlated with higher levels of owner affection and attachment towards their cat [94] although their style of attachment is more likely to be ‘anxious’ than ‘secure’ [94,155,156].

Whilst our data showed no significant differences in the source of cat acquisition based on owner personality, we identified a greater likelihood that neurotic owners would have a non-pedigree rather than pedigree cat. Exactly why this is the case remains entirely conjecture, but is worthy of speculation. It may be that owners are choosing cats based on the perceived complementarity or reciprocity of the cat’s behaviour, relative to their own personality. Evidence suggests that owner satisfaction is greater when their cat has a similar level of warmth to themselves, but a complementary level of dominance-submissiveness [157]. The finding that non-pedigree cats were reported as having less gregarious and more aggressive, aloof/avoidant and anxious/fearful behavioural styles within our study is potentially partially consistent with the similarity hypothesis regarding the relationship between neuroticism and non-pedigree selection. Interestingly, in dogs, whilst no relationship between owner Neuroticism and breed owned was found, owner Psychoticism was significantly higher in individuals owning breeds that may be commonly perceived as being more “aggressive” [158]. Thus, owner personality may be an important factor in breed selection, but that its effect may vary depending on the species in question.

Further studies are necessary in order to understand the causal mechanisms for the relationship between owner personality and cat behavioural styles as well as the drivers of pet selection. It is currently unknown as to whether the personality of the owner is directly influencing the behavioural responses of cats, whether neurotic owners are more likely to select cats whose behaviour they perceive as most similar to their own, and whether they may have a more pessimistic view of their cat’s behaviour.

Certain cat demographic and lifestyle features were also associated with different potential welfare outcomes for cats. In line with earlier research [25,108,159], older cats were significantly more likely to have lower sickness behaviour scores (indicating a greater frequency of sickness behaviours) and lower house soiling behaviour scores (indicating a greater frequency of house soiling). This indicates a degree of validity associated with the use of owner reports of cat health in this study. Interestingly, we did not find any direct relationship between cat behavioural styles and stress-linked sickness behaviours, although both were significantly and negatively related to owner Neuroticism. Such results might suggest that the sickness behaviours we measured are more a reflection of the impact of the environment on the cat (i.e. their management and owner Neuroticism), rather than a reflection of their individual variability in being able to cope. This also reflects the fact that different measures or indicators of potential stress do not always correlate e.g. [160].

Strictly indoor cats or those given restricted outdoor access only were more likely to display stress-linked sickness behaviours. However, in contrast to previous research, they were not more likely to be reported as overweight [107,137]. A higher reported incidence of sickness behaviours in indoor cats could simply reflect these owners being in a better position to monitor their cat for relevant signs of ill health (e.g. vomiting, diarrhoea, constipation, and cystitis). However, if this was the case, it would be reasonable to suppose that there should also be significant differences in health scores between cats kept strictly indoors and those allowed some form of restricted outdoor access. This was not the case. The lower prevalence of sickness behaviour found among cats given unrestricted outdoor access may thus suggest this lifestyle could be less stressful for cats, providing them with choice and control in relation to a potentially valuable resource. Evidence suggests that certain health risk factors may increase or decrease relative to the amount of time the cat has access to the outside [107]. Therefore, future work should consider a more sensitive measure of the quantity (and quality) of outdoor access provided to cats whose access is restricted. Additionally, because we cannot identify the direction of causality, it may also be the case that cats are more likely to be kept indoors by their owners if they begin to show sickness behaviours, as a way to more closely monitor their health.

Pedigree cats were more likely to be overweight than underweight, although they were not more likely to be overweight than of a normal weight, suggesting only a weak link between pedigree breeds and obesity risk. Indeed, previous research either finds no difference in obesity risk based on breed status [137] or a greater risk for non-pedigree cats in the US [107]. Pedigree cats were also found to be more likely to be kept strictly indoors or have restricted access to the outdoors than unrestricted access, a finding in concordance with previous studies [57,161]. Whilst pedigree cats were not more likely to display house soiling or sickness behaviours, they were found to be more likely to have a pre-existing medical condition, a finding also supported by previous evidence indicating certain pedigree breeds can be at greater risk of poor health [49,53,54,107]. Because the composite sickness parameter used was comprised of items chosen specifically for their links to stress rather than condition-specific behavioural signs of ill health or poor quality of life, it is not necessarily surprising that there were no significant differences in sickness scores relative to breed type.

Whilst various significant relationships between response and explanatory variables were indicated, estimates for fixed effects were generally relatively small compared to the intercepts in the various models (see S6 Appendix). Therefore given the relatively large size of the sample, and especially where in some cases the results were marginally significant, the relative effect at a population level may be conservative. Nonetheless, the identification of the presence of such relationships helps to elucidate the potential links between owner personality and care outcomes for cats, and is therefore an important initial exploratory investigation into this phenomenon.

We used a convenience, self-selecting sampling method, recruiting people via specific online platforms (predominantly social media). Due to various demographic biases associated with such types of sampling methods [162], it is important to be aware of the extent to which such groups of participants may or may not be representative of the UK cat owning public. To keep the amount of questions to a minimum, we collected only very basic demographic information from cat owners which did not include items relating to their socioeconomics, ethnicity or education levels. Therefore it was not possible to ascertain how these specific variables were distributed across the sample population. Our response rates were also heavily biased towards female participants. However, very similar response biases have been reported in similar types of online cat owner surveys conducted in the UK, US and also Australia [94,137,163–165], suggesting this may be a common phenomenon associated with these types of studies and sampling methods. Additionally, previous online pet-based surveys containing participants of both mixed and predominantly US nationality reported similar BFI personality scores [92,94] as well as standard deviations [94] to those found in the current study. Collectively, these cross-study similarities suggest that the current findings are potentially comparable in a broader research context.

Cat demographics from the current study such as average cat age, proportion of cats neutered, proportion of males versus females, proportion of domestic short hair versus pure bred, and source of cat acquisition were also generally similar with those reported elsewhere (based on surveys of UK cat owners [69,103,137,166,167]). The exception to this was in relation to outdoor access provision. In the current study, rates of cats being kept either solely indoors, or having restricted access to the outdoors, were slightly higher, and rates of unrestricted access slightly lower, compared to those documented elsewhere [137,166]. It is possible that this variability could simply reflect differences in sample sizes (the current study included more than three times as many respondents and may thus be more representative of UK cat owner practices). However, it is also likely that in addition to the owner’s personality, types of outdoor access provision are influenced by owner demographics such as housing type, location and local rates of road traffic accidents involving cats, and that such demographics may have varied between studies. Further research to investigate potential links between cat owner demographics and outdoor access provision would thus be beneficial.

## Conclusions

This study provides the best evidence to date of the relationship between owner personality and cat behaviour, welfare and lifestyle parameters, showing for the first time clear parallels with the parent-child relationship and the associated wellbeing outcomes for children. Our results present initial evidence which may indicate that lower levels of owner Neuroticism but higher levels of Agreeableness, Conscientiousness, Extroversion and Openness may potentially be generally more beneficial for cats; a finding mirroring conclusions from the parent-child literature [82]. Whilst various demographic and lifestyle features of the cat were found to interact with their various health and behavioural parameters (and therefore could act as potential confounds when exploring relationships between owner personality and these variables), their inclusion as random effects where appropriate in the relevant statistical models ensured that their effects were suitably controlled, allowing the relationship between owner personality and cat behaviour, management and health outcomes to be more clearly identified. However, it is important to iterate that parameters relating to the cat’s health and behaviour were derived solely from owner reports and therefore may be subject to various forms of reporting bias which are influenced by other demographic features of the owner.

In order to obtain a clearer picture of the underpinning mechanisms driving the relationship between owner personality and aspects of cat wellbeing, future important steps will be to ascertain (i) if and how owner personality influences the selection of cats with more health and behavioural problems and certain personality attributes (ii) how owner personality is related to the nature of owner-cat interactions and husbandry, and (iii) how behavioural and biological measures collected directly from the cat relate to owner reports of cat wellbeing and owner personality.

## Supporting information

S1 Appendix Cat owner questionnaire. Items included are relevant to all variables and analyses conducted within the paper. Questionnaire was available online via Survey Monkey from June-July of 2016.
(DOCX)

S2 Appendix Cat behavioural profiles. Profiles based on PC loadings for owner reported cat behaviour. All items retained loaded at ≥ |0.4|. Negative loadings for questions are indicated in bold.
(DOCX)

S3 Appendix Demographic features of the cat owner population and relevant descriptive statistics. (DOCX)

S4 Appendix Demographic features of the cat population and relevant descriptive statistics. (DOCX)

S5 Appendix Questionnaire data. (DOCX)

S6 Appendix Summary outputs from GLM and GLMM minimum adequate models. (DOCX)
