# Supplementary material for: Owner personality and the wellbeing of their cats share parallels with the parent-child relationship
Source: PLoS One. 2019 Feb 5;14(2):e0211862. doi: 10.1371/journal.pone.0211862 (PMC6363285; doi:10.1371/journal.pone.0211862)
Supplement: S1 Appendix — Items included are relevant to all variables and analyses conducted within the paper. Questionnaire was available online via Survey Monkey from June-July of 2016. (DOCX) [file pone.0211862.s001.docx]

Thank you for agreeing to participate in this study. This questionnaire is part of an MSc study being carried out at the University of Lincoln. Its aim is to investigate the relationship between owner personality and cat-human interactions. All responses are confidential and anonymous and will only be used in relation to this research. This first section is about your cat. If you have more than one cat, please choose the cat you feel you know best.

| Age of cat |  | | | | | | | | | | |
| --- | --- | --- | --- | --- | --- | --- | --- | --- | --- | --- | --- |
| Is your cat male or female? | Male ⃝ | | | Female ⃝ | |  | | |  | | |
| Where did you get your cat from? | Rehoming centre ⃝ | | | Breeder ⃝ | | Friend/Neighbour ⃝ | | | Other ____________ | | |
| Is your cat neutered? | Yes ⃝ | | | No ⃝ | | Unsure ⃝ | | |  | | |
| Breed of cat | Domestic short/long hair ⃝ Other ⃝  Please provide the details of your cat’s breed _____________________________ | | | | | | | | | | |
| Does your cat have regular access to the outdoors | Yes ⃝ | | | No ⃝ | |  | | |  | | |
| If yes, the access controlled by yourself (i.e. is your cat kept indoors at night | Constant access ⃝ | | | Restricted access ⃝ | |  | | |  | | |
| Does your cat have any pre-existing medical conditions | Yes ⃝ | | | No ⃝ | |  | | |  | | |
| If yes, what condition does he/she suffer from? |  | | | | | | | | | | |
| Does your cat display any behavioural problems? | Yes ⃝ | | No ⃝ | | | |  | | |  | |
| If yes, what behavioural problem does he/she display? |  | | | | | | | | | | |
|  |  | | | | | | | | | | |
| Does your cat suffer from a urinary tract infection (e.g. cystitis)? | Always ⃝ Often ⃝ Occasionally ⃝ Once ⃝ Never ⃝ | | | | | | | | | | |
| Does your cat urinate inside the house in a location other than the litter tray? | Always ⃝ Often ⃝ Occasionally ⃝ Once ⃝ Never ⃝ | | | | | | | | | | |
| Does your cat spray urine on any vertical surfaces? (e.g. curtains, kitchen cabinets, doors, walls) | Always ⃝ Often ⃝ Occasionally ⃝ Once ⃝ Never ⃝ | | | | | | | | | | |
| Does your cat defecate inside the house in a location other than the litter tray? | Always ⃝ Often ⃝ Occasionally ⃝ Once ⃝ Never ⃝ | | | | | | | | | | |
| Does your cat vomit? | Always ⃝ Often ⃝ Occasionally ⃝ Once ⃝ Never ⃝ | | | | | | | | | | |
| Does your cat suffer from diarrhoea? | Always ⃝ Often ⃝ Occasionally ⃝ Once ⃝ Never ⃝ | | | | | | | | | | |
| Does your cat suffer from constipation? | Always ⃝ Often ⃝ Occasionally ⃝ Once ⃝ Never ⃝ | | | | | | | | | | |
| How would you describe your cat’s weight? | Very overweight ⃝ Overweight ⃝ Normal ⃝ Underweight ⃝ Very underweight ⃝ | | | | | | | | | | |
| What is the condition of your cat’s coat? | ⃝  1  Dirty and greasy, lots of mats, lots of bald patches | ⃝  2  Always has a few greasy patches, Usually has at least one mat, more than 1 bald patch | | | ⃝  3  Some greasy patches, occasional mats, 1 bald patch | | | ⃝  4  Usually has shiny and silky coat but has had the occasional greasy patch, small bald spot or mat | | | ⃝  5  Shiny and silky, no mats or bald spots |

Please answer these questions about your cat’s behaviour (please ensure this is the same cat that you chose to answer the above questions for)

1. My cat is keen to explore new things in its environment

Never ⃝ Once ⃝ Occasionally ⃝ Usually ⃝ Always ⃝

1. My cat is playful

Strongly agree ⃝ Agree ⃝ Neither agree nor disagree ⃝ Disagree ⃝ Strongly disagree ⃝

1. My cat is vocal when around people:

Never ⃝ Once ⃝ Occasionally ⃝ Usually ⃝ Always ⃝

1. My cat is comfortable being picked up

Strongly agree ⃝ Agree ⃝ Neither agree nor disagree ⃝ Disagree ⃝ Strongly disagree ⃝

1. My cat tries to avoid me when I try to encourage interaction (i.e. – when I call it’s name in a friendly voice, when I make kissing noises, or crouch down and offer it my fingers, etc.)

Never ⃝ Once ⃝ Occasionally ⃝ Usually ⃝ Always ⃝

1. My cat tries to avoid me when I go to stroke it or tickle its chin/cheeks:

Never ⃝ Once ⃝ Occasionally ⃝ Usually ⃝ Always ⃝

1. When I initiate contact or interaction with my cat, it doesn’t move away but it is quiet and not very responsive towards me (i.e. it doesn’t purr or rub up against me):

Never ⃝ Once ⃝ Occasionally ⃝ Usually ⃝ Always ⃝

1. My cat is timid

Strongly agree ⃝ Agree ⃝ Neither agree nor disagree ⃝ Disagree ⃝ Strongly disagree ⃝

1. My cat will come and say ‘hello’ and approach me (i.e. the cat will approach and make physical contact with me), but will then wander off or move away shortly afterwards rather than staying for a long fuss:

Never ⃝ Once ⃝ Occasionally ⃝ Usually ⃝ Always ⃝

1. My cat comes and asks me for attention and initiates contact with me (e.g. the cat comes and sits on my knee, or rubs up against me and around me, in order to receive fuss/strokes/chin/cheek tickles):

Never ⃝ Once ⃝ Occasionally ⃝ Usually ⃝ Always ⃝

1. My cat behaves aggressively (i.e. growls, hisses, bites, swipes with claws) towards me when I stroke it:

Never ⃝ Once ⃝ Occasionally ⃝ Usually ⃝ Always ⃝

1. My cat behaves aggressively (i.e. growls hisses, bites, swipes with claws) towards me when I perform routine health procedures (such as grooming/carrying out health checks, or when administering medication, etc.)

Never ⃝ Once ⃝ Occasionally ⃝ Usually ⃝ Always ⃝

1. My cat is quick to settle and to adapt to change

Strongly agree ⃝ Agree ⃝ Neither agree nor disagree ⃝ Disagree ⃝ Strongly disagree ⃝

1. My cat gets carried away during play, which has led me to being bitten or swiped at:

Never ⃝ Once ⃝ Occasionally ⃝ Usually ⃝ Always ⃝

1. My cat would prefer to be left alone, rather than be with people:

Strongly agree ⃝ Agree ⃝ Neither agree nor disagree ⃝ Disagree ⃝ Strongly disagree ⃝

1. My cat likes being stroked:

Strongly agree ⃝ Agree ⃝ Neither agree nor disagree ⃝ Disagree ⃝ Strongly disagree ⃝

1. I avoid stroking or handling my cat because I feel that it doesn’t want me to:

Never ⃝ Once ⃝ Occasionally ⃝ Usually ⃝ Always ⃝

1. My cat is very tolerant to being handled:

Strongly agree ⃝ Agree ⃝ Neither agree nor disagree ⃝ Disagree ⃝ Strongly disagree ⃝

1. I avoid stroking my cat because I think it will behave aggressively towards me (i.e. growl, hiss, bite, swipe with claws)

Never ⃝ Once ⃝ Occasionally ⃝ Usually ⃝ Always ⃝

1. My cat seems angry around me:

Never ⃝ Once ⃝ Occasionally ⃝ Usually ⃝ Always ⃝

1. If my cat could chose, it would prefer to have a bowl of food rather than interact with me:

Strongly agree ⃝ Agree ⃝ Neither agree nor disagree ⃝ Disagree ⃝ Strongly disagree ⃝

1. My cat is more keen to interact with and be near me when I have food/treats:

Never ⃝ Once ⃝ Occasionally ⃝ Usually ⃝ Always ⃝

1. My cat has negatively changed in the way it interacts with me since I first acquired him/her (e.g. has become more fearful, behaves more aggressively, is less friendly)

Strongly agree ⃝ Agree ⃝ Neither agree nor disagree ⃝ Disagree ⃝ Strongly disagree ⃝

1. My cat has positively changed in the way it interacts with me since I first acquired him/her (e.g. has become less fearful, behaves less aggressively, is more friendly)

Strongly agree ⃝ Agree ⃝ Neither agree nor disagree ⃝ Disagree ⃝ Strongly disagree ⃝

1. My cat behaves differently with strangers than he/she does with me:

Strongly agree ⃝ Agree ⃝ Neither agree nor disagree ⃝ Disagree ⃝ Strongly disagree ⃝

1. My cat behaves differently with me than he/she does with other (human) members of the household:

Strongly agree ⃝ Agree ⃝ Neither agree nor disagree ⃝ Disagree ⃝ Strongly disagree ⃝

1. My cat is friendly

Strongly agree ⃝ Agree ⃝ Neither agree nor disagree ⃝ Disagree ⃝ Strongly disagree ⃝

1. My cat is fearful

Strongly agree ⃝ Agree ⃝ Neither agree nor disagree ⃝ Disagree ⃝ Strongly disagree ⃝

1. My cat has met all my expectations

Strongly agree ⃝ Agree ⃝ Neither agree nor disagree ⃝ Disagree ⃝ Strongly disagree ⃝

1. I am happy with my cat:

Strongly agree ⃝ Agree ⃝ Neither agree nor disagree ⃝ Disagree ⃝ Strongly disagree ⃝

1. I feel my cat is happy living with me:

Strongly agree ⃝ Agree ⃝ Neither agree nor disagree ⃝ Disagree ⃝ Strongly disagree ⃝

1. I have considered relinquishing or rehoming this cat to someone else

Never ⃝ Once ⃝ Occasionally ⃝ Usually ⃝ Always ⃝

Thank you for completing the first part of this questionnaire. We now need a few basic details about yourself.

| Gender | Male ⃝ | Female ⃝ | Prefer not to say ⃝ |  |
| --- | --- | --- | --- | --- |
| Age | 18-24 ⃝ | 25-34 ⃝ | 35-44 ⃝ | 45-54 ⃝ |
|  | 55-64 ⃝ | Over 65 ⃝ |  |  |
| Number of other cats in the household |  |  |  |  |

Please answer these questions about yourself, picking the answer that first comes to you.

| I see myself as someone who.... | Disagree strongly | Disagree a little | Neither agree nor disagree | Agree a little | Agree strongly |
| --- | --- | --- | --- | --- | --- |
| 1. Is talkative | ⃝ | ⃝ | ⃝ | ⃝ | ⃝ |
| 1. Tends to find fault with others | ⃝ | ⃝ | ⃝ | ⃝ | ⃝ |
| 1. Does a thorough job | ⃝ | ⃝ | ⃝ | ⃝ | ⃝ |
| 1. Is depressed, blue | ⃝ | ⃝ | ⃝ | ⃝ | ⃝ |
| 1. Is original, comes up with new ideas | ⃝ | ⃝ | ⃝ | ⃝ | ⃝ |
| 1. Is reserved | ⃝ | ⃝ | ⃝ | ⃝ | ⃝ |
| 1. Is helpful and unselfish with others | ⃝ | ⃝ | ⃝ | ⃝ | ⃝ |
| 1. Can be somewhat careless | ⃝ | ⃝ | ⃝ | ⃝ | ⃝ |
| 1. Is relaxed, handles stress well | ⃝ | ⃝ | ⃝ | ⃝ | ⃝ |
| 1. Is curious about many different things | ⃝ | ⃝ | ⃝ | ⃝ | ⃝ |
| 1. Is full of energy | ⃝ | ⃝ | ⃝ | ⃝ | ⃝ |
| 1. Starts quarrels with others | ⃝ | ⃝ | ⃝ | ⃝ | ⃝ |
| 1. Is a reliable worker | ⃝ | ⃝ | ⃝ | ⃝ | ⃝ |
| 1. Can be tense | ⃝ | ⃝ | ⃝ | ⃝ | ⃝ |
| 1. Is ingenious, a deep thinker | ⃝ | ⃝ | ⃝ | ⃝ | ⃝ |
| 1. Generates a lot of enthusiasm | ⃝ | ⃝ | ⃝ | ⃝ | ⃝ |
| 1. Has a forgiving nature | ⃝ | ⃝ | ⃝ | ⃝ | ⃝ |
| 1. Tends to be disorganised | ⃝ | ⃝ | ⃝ | ⃝ | ⃝ |
| 1. Worries a lot | ⃝ | ⃝ | ⃝ | ⃝ | ⃝ |
| 1. Has an active imagination | ⃝ | ⃝ | ⃝ | ⃝ | ⃝ |
| 1. Tends to be quiet | ⃝ | ⃝ | ⃝ | ⃝ | ⃝ |
| 1. Is generally trusting | ⃝ | ⃝ | ⃝ | ⃝ | ⃝ |
| 1. Tends to be lazy | ⃝ | ⃝ | ⃝ | ⃝ | ⃝ |
| 1. Is emotionally stable, not easily upset | ⃝ | ⃝ | ⃝ | ⃝ | ⃝ |
| 1. Is inventive | ⃝ | ⃝ | ⃝ | ⃝ | ⃝ |
| 1. Has an assertive personality | ⃝ | ⃝ | ⃝ | ⃝ | ⃝ |
| 1. Can be cold and aloof | ⃝ | ⃝ | ⃝ | ⃝ | ⃝ |
| 1. Perseveres until the task is finished | ⃝ | ⃝ | ⃝ | ⃝ | ⃝ |
| 1. Can be moody | ⃝ | ⃝ | ⃝ | ⃝ | ⃝ |
| 1. Values artistic, aesthetic experiences | ⃝ | ⃝ | ⃝ | ⃝ | ⃝ |
| 1. Is sometimes shy, inhibited | ⃝ | ⃝ | ⃝ | ⃝ | ⃝ |
| 1. Is considerate and kind to almost everyone | ⃝ | ⃝ | ⃝ | ⃝ | ⃝ |
| 1. Does things efficiently | ⃝ | ⃝ | ⃝ | ⃝ | ⃝ |
| 1. Remains calm in tense situations | ⃝ | ⃝ | ⃝ | ⃝ | ⃝ |
| 1. Prefers work that is routine | ⃝ | ⃝ | ⃝ | ⃝ | ⃝ |
| 1. Is outgoing, sociable | ⃝ | ⃝ | ⃝ | ⃝ | ⃝ |
| 1. Is sometimes rude to others | ⃝ | ⃝ | ⃝ | ⃝ | ⃝ |
| 1. Makes plans and follows through with them | ⃝ | ⃝ | ⃝ | ⃝ | ⃝ |
| 1. Gets nervous easily | ⃝ | ⃝ | ⃝ | ⃝ | ⃝ |
| 1. Likes to reflect, play with ideas | ⃝ | ⃝ | ⃝ | ⃝ | ⃝ |
| 1. Has few artistic interests | ⃝ | ⃝ | ⃝ | ⃝ | ⃝ |
| 1. Likes to cooperate with others | ⃝ | ⃝ | ⃝ | ⃝ | ⃝ |
| 1. Is easily distracted | ⃝ | ⃝ | ⃝ | ⃝ | ⃝ |
| 1. Is sophisticated in art, music or literature | ⃝ | ⃝ | ⃝ | ⃝ | ⃝ |

Thank you for taking the time to complete this study. If you would like to receive a short summary of the research once it is complete then please leave your e-mail address. Your e-mail address will only be used to receive the research summary and will be kept confidentially.

E-mail: ­­­­­­­­­______________________
