# Supplementary material for: Owner personality and the wellbeing of their cats share parallels with the parent-child relationship
Source: PLoS One. 2019 Feb 5;14(2):e0211862. doi: 10.1371/journal.pone.0211862 (PMC6363285; doi:10.1371/journal.pone.0211862)
Supplement: S2 Appendix — Profiles based on PC loadings for owner reported cat behaviour. All items retained loaded at ≥ |0.4|. Negative loadings for questions are indicated in bold. (DOCX) [file pone.0211862.s002.docx]

| **PC1: Gregariousness** (confident, friendly, interactive) | **PC2: Aggressiveness** (agonistic when handled or interacted with) | **PC3: Aloofness/avoidance** (avoidant of interaction, not friendly) | **PC4: Anxiousness/fearfulness** (wary of novel/less familiar people) |
| --- | --- | --- | --- |
| My cat is keen to explore new things in his/her environment | My cat behaves aggressively (i.e. growls, hisses, bites, swipes with claws) towards me when I perform routine health procedures (such as grooming/carrying out health checks, or when administering medication, etc.) | My cat tries to avoid me when I try to encourage interactions (i.e. when I call his/her name in a friendly voice, when I make kissing noises, or crouch down and offer it my fingers etc.) | My cat is timid |
| My cat is playful | My cat behaves aggressively (i.e. growls, hisses, bites and swipes with claws) towards me when I stroke him/her | My cat tries to avoid me when I go to stroke him/her or tickle his/her chin/cheeks | My cat has positively changed in the way he/she interacts with me since I first acquired him/her (e.g. has become less fearful, behaves less aggressively, is more friendly) |
| My cat is vocal when around people | My cat gets carried away during play, which has led me to being bitten or swiped | When I initiate contact or interaction with my cat, he/she doesn't move away but it is quiet and not very responsive towards me (i.e. it doesn't purr or rub up against me) | My cat behaves differently with strangers than he/she does with me |
| My cat comes and asks me for attention and initiates contact with me (e.g. the cat comes and sits on my knee, or rubs up against me and around me, in order to receive fuss/strokes/chin/cheek tickles) | I avoid stroking or handling my cat because I feel that he/she doesn't want me to | My cat will come and say "hello" and approach me (i.e. the cat will approach and make physical contact with me), but will then wander off or move away shortly afterwards rather than staying for a long fuss | My cat behaves differently with me than he/she does with other (human) members of the household |
| My cat is comfortable being picked up | I avoid stroking my cat because I think he/she will behave aggressively towards me (i.e. growl, hiss, bite, swipe with claws) | **My cat comes and asks me for attention and initiates contact with me (e.g. the cat comes and sits on my knee, or rubs up against me and around me, in order to receive fuss/strokes/chin/cheek tickles)** | My cat is fearful |
| **My cat is timid** | My cat seems angry around me | My cat is more keen to interact with and be near me when I have food/treats |  |
| My cat is quick to settle and to adapt to change | **My cat is very tolerant to being handled** | **My cat likes being stroked** |  |
| **My cat would prefer to be left alone, rather than be with people** |  | If my cat could choose, it would prefer to have a bowl of food rather than interaction with me |  |
| My cat is very tolerant to being handled |  |  |  |
| My cat is friendly |  |  |  |
| **My cat is fearful** |  |  |  |
