# Supplementary material for: Owner personality and the wellbeing of their cats share parallels with the parent-child relationship
Source: PLoS One. 2019 Feb 5;14(2):e0211862. doi: 10.1371/journal.pone.0211862 (PMC6363285; doi:10.1371/journal.pone.0211862)
Supplement: S3 Appendix — (DOCX) [file pone.0211862.s003.docx]

| **Cat owner demographics:** | | | | | | | |
| --- | --- | --- | --- | --- | --- | --- | --- |
| Gender | Female: 2923 (92%) | | Male: 233 (7%) | | | Prefer not to say: 19 (0.6%) | |
| Age | 18-24: 305 (9.6%) | 25-34:  819 (25.9%) | 35-44:  819 (25.9%) | | 45-54:  735 (23.2%) | 55-64:  347 (11%) | 65+:  113 (3.6%) |
| Additional cats owned | Mean: 1.4 (± 2.2sd) Median: 1 Min: 0 Max: 32 | | | | | | |
| Source of cat acquisition | Online/Advert/pet shop: 185 (5.8%) | | Bred from/born at home:  72 (2.3%) | | | Breeder:  430 (13.6%) | |
|  | Stray/abandoned: 306 (9.7%) | | Family/friend/acquaintance: 825 (26.1%) | | | Feral/from a farm:  63 (2%) | |
|  | Veterinary clinic: 142 (4.5%) | | Rescue or rehoming organisation/foster group/sanctuary: 1098 (34.7%) | | | Other: 44 (1.4%) | |
| Outdoor access provision | Constant outdoor access: 916 (28.9%) | | Restricted outdoor access: 1423 (45%) | | | Strictly indoors:  826 (26.1%) | |
| **Owner personality scores based on the BFI (John et al 1991)** | | | | | | | |
| Agreeableness score | Mean: 34.7 (± sd 5.5) Median: 35 Min: 10 Max: 45  Scale range: 9 – 45  Average scale score:  Mean: 3.85 (± sd 0.61) | | | Composite measures based on the average of the sum of 8-10 items rated on a 5 point Likert scale from Disagree strongly to Agree strongly. Certain items reversed scored so that high scores always reflect a high expression of relevant trait. See (John et al 1991) for full list of items and their scoring method | | | |
| Conscientiousness score | Mean: 32.68 (± sd 3.9) Median: 33 Min: 17 Max: 43  Scale range: 9 – 45  Average scale score:  Mean: 3.631 (± sd 0.43) | | |  |  |  |  |
| Extraversion score | Mean: 25.79 (± sd 4.9) Median:26 Min: 11 Max: 39  Scale range: 8- 40  Average scale score:  Mean: 3.22 (± sd 0.61) | | |  |  |  |  |
| Openness score | Mean: 37.54 (± sd 5.8) Median: 38 Min: 20 Max: 50  Scale range: 10-50  Average scale score:  Mean: 3.75 (± sd 0.58) | | |  |  |  |  |
| Neuroticism score | Mean: 24.12 (± sd 6.7) Median: 24 Min: 8 Max: 40  Scale range: 8 –40  Average scale score:  Mean: 3.01 (± sd 0.84) | | |  |  |  |  |
| **Owner satisfaction** | | | | | | | |
| Satisfaction with cat | Mean: 14.12(± sd 1.5) Median: 15 Min: 3 Max: 15  Scale range 3-15 | | | Composite measure derived from sum of 3 items, rated on a 5-point Likert scale representing agreement with statements about the cat from Strongly disagree (1) to Strongly agree (5) | | | |
| Consideration of relinquishing cat | Mean:1.1 (±sd 0.5) Median: 1 Min: 1 Max: 5  Scale range 1-5 | | | Rated on a 5-point Likert scale based on frequency of considering relinquishment from Never (1) to Always (5) | | | |
