# Supplementary material for: Owner personality and the wellbeing of their cats share parallels with the parent-child relationship
Source: PLoS One. 2019 Feb 5;14(2):e0211862. doi: 10.1371/journal.pone.0211862 (PMC6363285; doi:10.1371/journal.pone.0211862)
Supplement: S4 Appendix — (DOCX) [file pone.0211862.s004.docx]

| **Cat demographics** | | | | | |
| --- | --- | --- | --- | --- | --- |
| Breed | Non-pedigree (including domestic short, medium and long haired cats):  2612 (82.5%) | Pedigree: 553 (17.5%) | Pedigree cats included the following breeds and their crosses:  Main coon, Turkish Van, Sphinx, Tonkinese, Toyger, Siamese, Serengeti, Savannah, Russian blue/white, Ragdoll, Persian, Scottish fold, Bengal, Burmese, Asian, Abyssinian, American shorthair, Balinese, Turkish angora, American curl, Birman, Bombay, Oriental, British blue, Aphrodite giant | | |
| Age | Mean: 7.194 (± sd 4.9) Median: 6 Min: < 1 Max: 24 | | | | |
| Neuter status | Neutered:  3077 (97.2%) | | Unneutered:  75 (2.4%) | | Unsure:  13 (0.4%) |
| **Cat health parameters as rated by owner** | | | | | |
| Pre-existing Medical condition | No:  2423 (76.6%) | Yes:  742 (23.4%) | Medical conditions included: Kidney disease, gingivitis and teeth problems, asthma and respiratory problems, cataracts, URI’s , Chronic diarrhoea, chronic ear infections, cardiomyopathy, cancer, tumours, blocked anal glands, blocked tear ducts, blindness, deafness, joint problems and dysplasia, arthritis, chronic cystitis, bladder stones/crystal, hypo/hyperthyroidism, diabetes, cerebellar hypoplasia, hypertension, food intolerance, heart murmur, flea allergy, hernia, gastrointestinal problems, limb amputations, FIV+, Felv+, renal failure, epilepsy, dementia, amyloidosis | | |
| Sickness behaviour scores | Mean: 22.11 (± sd 2) Median: 22  Min: 14 Max: 25  Scale range: | | Composite measure derived from sum of 5 items, 4 based on frequency (from Never (5) to always (1)) ratings of behaviours relating to vomiting, diarrhoea, constipation, cystitis and one item relating to coat condition  (rated from poor condition (1) to excellent (5)) | | |
| House soiling scores | Mean: 14 (± sd 1.6) Median: 15  Min: 3 Max: 15  Scale range: 1- 15 | | Composite measure derived from sum of 3 items based on frequency ratings of house soiling behaviours (spraying and urination and defecation outside of the litter tray) from Never (5) to always (1) | | |
| Weight category | Normal | Overweight or very overweight | Underweight or very underweight | Initially comprising of five separate categories, two of which were then collapsed based on lack of responses for the extreme weight categories | |
|  | 2401(76%) | 552 (17%) | 211 (7%) |  |  |
| **Cat behaviour as rated by their owner** | | | | | |
| Behavioural problem | No 2542 (80%) | Yes 623 (20%) | Behavioural problems mentioned included:  Fear/anxiety  House soiling  Aggression (inter and intraspecific or unspecified)  Over grooming  Avoidance of people  Excessive vocalisations  Scratching on furniture  Separation anxiety  Obsessive compulsive Disorder  Attention seeking  Cognitive dysfunction | | |
| PC1: ‘Gregariousness’ | Mean: 37.8 (± 6.4sd)  Median:38  Min: 12  Max: 50  Scale range: 10-50 | Composite measures based on the sum of question items (from 5-10), rated on 5-point Likert scales representing agreement with a statement about the cat (from Strongly disagree (1) to Strongly agree (5) or the frequency of a behaviour occurrence (from Never to Always). Certain items reversed scored so that high scores always reflect a high expression of relevant trait. See Table 7 of appendix for full list of items and their inclusion in each of the retained components. | | | |
| PC2: ‘Aggressiveness’ | Mean: 11.32(±4 sd)  Median: 10  Min: 7  Max: 33  Scale range: 7-35 |  |  |  |  |
| PC3: ‘Aloofness/avoidance’ | Mean: 15.22(± 4.6sd)  Median: 14  Min: 8  Max:39  Scale range: 8- 40 |  |  |  |  |
| PC4: ‘Anxiousness/fearfulness’ | Mean: 15.98(±3.7 sd)  Median:16  Min: 5  Max:25  Scale range: 5-25 |  |  |  |  |
